# Dexmedetomidine Attenuates Ferroptosis-Mediated Renal Ischemia/Reperfusion Injury and Inflammation by Inhibiting ACSL4 *via* α2-AR

**DOI:** 10.3389/fphar.2022.782466

**Published:** 2022-06-14

**Authors:** Wen-hui Tao, Xi-sheng Shan, Jia-xin Zhang, Hua-yue Liu, Bi-ying Wang, Xiang Wei, Mian Zhang, Ke Peng, Jun Ding, Shang-xian Xu, Lin-gui Li, Jun-kai Hu, Xiao-wen Meng, Fu-hai Ji

**Affiliations:** ^1^ Department of Anesthesiology, First Affiliated Hospital of Soochow University, Soochow, China; ^2^ Institute of Anesthesiology, Soochow University, Soochow, China

**Keywords:** Dexmedetomidine, Renal ischemia/reperfusion injury, ferroptosis, Inflammation, ACSL4, α2-adrenergic receptor

## Abstract

Ischemia-reperfusion (I/R) injury is a serious clinical pathology associated with acute kidney injury (AKI). Ferroptosis is non-apoptotic cell death that is known to contribute to renal I/R injury. Dexmedetomidine (Dex) has been shown to exert anti-inflammatory and organ protective effects. This study aimed to investigate the detailed molecular mechanism of Dex protects kidneys against I/R injury through inhibiting ferroptosis. We established the I/R-induced renal injury model in mice, and OGD/R induced HEK293T cells damage *in vitro*. RNA-seq analysis was performed for identifying the potential therapeutic targets. RNA-seq analysis for differentially expressed genes (DEGs) reported Acyl-CoA synthetase long-chain family member 4 (ACSL4) related to ferroptosis and inflammation in I/R mice renal, which was validated in rodent renal. Liproxstatin-1, the specific small-molecule inhibitor of ferroptosis, significantly attenuated ferroptosis-mediated renal I/R injury with decreased LPO, MDA, and LDH levels, and increased GSH level. Inhibiting the activity of ACSL4 by the Rosiglitazone (ROSI) resulted in the decreased ferroptosis and inflammation, as well as reduced renal tissue damage, with decreasing LPO, MDA and LDH level, increasing GSH level, reducing COX2 and increasing GPx4 protein expression, and suppressing the TNF-α mRNA and IL-6 mRNA levels. Dex as a α2-adrenergic receptor (α2-AR) agonist performed renal protective effects against I/R-induced injury. Our results also revealed that Dex administration mitigated tissue damage, inhibited ferroptosis, and downregulated inflammation response following renal I/R injury, which were associated with the suppression of ACSL4. In addition, ACSL4 overexpression abolishes Dex-mediated protective effects on OGD/R induced ferroptosis and inflammation in HEK293T cells, and promotion of ACSL4 expression by α2-AR inhibitor significantly reversed the effects on the protective role of Dex. This present study indicated that the Dex attenuates ferroptosis-mediated renal I/R injury and inflammation by inhibiting ACSL4 *via* α2-AR.

## 1 Introduction

Ischemia-reperfusion (I/R) injury is a common clinical pathology associated with high mortality, occurred in numerous clinical pathologies, such as acute kidney injury (AKI) (Wang et al., 2020b), renal transplantation (Minami et al., 2020), and trauma shock (Hoareau et al., 2019), which leads to systemic inflammation and dysfunction (Hasegawa et al., 2021). Despite several mechanisms of I/R injury have been investigated, the effective treatment remains elusive. Therefore, it is meaningful to develop the potent therapeutic intervention for renal I/R injury.

Recently, increasing studies revealed that a variety of complex pathways were involved in renal I/R injuries, such as apoptosis (Liu et al., 2020), pyroptosis (Xia et al., 2021), and ferroptosis (Ding et al., 2020; Zhang et al., 2021). Attempts to prevent cell death have been strongly biased to the study of apoptosis for it has been considered to be the only form of cell death that can be reversed by pharmacological and genetic intervention. With the acquiring of a better understanding of cell death, ferroptosis has attracted extensive attention due to it is a novel type of cell death caused by iron-dependent phospholipid peroxidation (Doll and Conrad, 2017), which has been confirmed to be related to various diseases such as cancer (Wu et al., 2021b), degenerative diseases (Tang et al., 2021a), antiviral immunity (Xu et al., 2018), stroke (Alim et al., 2019), and I/R injury (Chen et al., 2021). Ferroptosis plays an important role in cisplatin-induced AKI (Hu et al., 2020). In addition, ferroptosis is involved in the AKI model induced by folic acid (Li et al., 2021). The study also found that isoliquiritigenin alleviates septic-induced AKI by suppressing ferroptosis (Tang et al., 2021b). However, the molecular mechanism of AKI is largely unknown. Now, with the direct link between lipid peroxidation and unique cell death pathway, the research on finding new small molecules that can inhibit lipid peroxidation has made progress, and may produce new cell protection strategies.

Ferroptosis is characterized by mitochondrial shrinkage, increased mitochondrial membrane density, and lipid reactive oxygen species (L-ROS), and a unique set of genes, such as Acyl-CoA synthetase long-chain family member 4 (ACSL4), Glutathione peroxidase (GPx4), and Cyclooxygenase2 (COX2) (Li et al., 2019). Lipid peroxidation is essential for ferroptosis and involves the preferential oxidation of arachidonic acid (AA) and its esterifiable production phosphatidylethanolamine (PE) (Lee et al., 2021). One study found that L-ROS inhibitors significantly alleviated myocardial I/R injury through inhibiting glutaminolysis and ferroptosis (Feng et al., 2019). Other studies demonstrated that ACSL4 could facilitate the esterification of AA and adrenoyl into PE, a process closely related to ferroptosis (Doll et al., 2017; Kagan et al., 2017), and suppression of this process by pharmacological ACSL4 inhibition induced anti-ferroptosis and anti-inflammation rescue pathway (Cui et al., 2021). However, the role of ferroptosis and inflammation in renal I/R is still not fully explored and remains elusive.

Dexmedetomidine (Dex) as a highly selective α2-adrenoceptor (AR) agonist alleviates septic heart injury by inhibiting ferroptosis (Wang et al., 2020a), and protects nerve cells from oxidative injury by maintaining iron homeostasis (Qiu et al., 2020). In addition, some studies have found that Dex could improve renal function and reduce I/R injury (Li et al., 2020a; Ma et al., 2020). However, the exact protective mechanism of Dex on renal I/R has not been elucidated. In this study, we hypothesize that Dex could protect the kidney against I/R induced injury through alleviating ferroptosis and inflammation by inhibiting ACSL4 *via* α2-AR.

## 2 Materials and Methods

### 2.1. Animals

Adult healthy C57BL/6 mice (7–8 weeks, 18–22 g) were obtained from Cavens Biogle Model Animal Research Co., Ltd. (Soochow, China). All mice were kept under controlled temperature of 24–26°C, relative humidity 40–60% and 12 h/12 h light-dark cycle with food and water available ad libitum. Animal care and handling were approved by the Institutional Animal Care and Use Committee of Soochow University (Soochow, China). All experiments were performed in accordance with the Guide for the Care of Use of Laboratory Animals published by the US National Institute of Health.

### 2.2 RNA Extraction, Library Construction and Sequencing

Total RNA was extracted employing Trizol reagent kit (Invitrogen, United States). Then, mRNA was enriched and fragmented and reverse transcripted into cDNA. After second-strand cDNA were synthesized, purified cDNA with QiaQuick PCR extraction kit (Qiagen, Netherlands), end repaired, poly (A) added, and ligated to Illumina sequencing adapters. PCR amplified, and sequenced using Illumina HiSeq2500 by Gene Denovo Biotechnology Co., (Guangzhou, China). FPKM (fragment per kilobase of transcript per million mapped reads) was calculated using StringTie software. RNAs differential expression analysis was performed by DESeq2 (Love, Huber and Anders 2014) between two different groups and by edgeR (Robinson, McCarthy and Smyth 2010) between two samples. The genes/transcripts with the parameter of false discovery rate (FDR) below 0.05 and absolute fold change ≥2 were considered differentially expressed genes/transcripts.

### 2.3. Establishment of Renal Ischemia-Reperfusion Model, Cell Culture, OGD/R Procedures and Transient Transfection

The renal I/R model was established as previous described (Liu et al., 2019). Briefly, renal I/R was induced by occluding bilateral renal hilum for 45 min by a microvascular clip and followed by 48 h reperfusion. Human Embryonic Kidney (HEK) 293T cells (ATCC, Rockville, United States) were grown in Dulbecco’s modified Eagle’s medium (DMEM), supplemented with 10% fetal bovine serum and 1% penicillin/streptomycin. Cells were incubated at 37°C in an atmosphere of 5% CO_2_, and grown to 80% confluence before use. For oxygen glucose deprivation/reoxygenation (OGD/R) treatment is the same as the description of our previous research (Yang et al., 2021b). Briefly, HEK293T were cultured in an anaerobic chamber containing 95% N_2_ and 5% CO_2_ for 6 h and then reoxygenated for 6 h to induce OGD/R condition. Cells were pretreated with Dex (1 μM, 24 h incubation before OGD/R) or/and ACSL4 overexpression vector transfection before exposure to OGD/R, as mentioned in this study.

The plasmid was created and synthesized by RiboBio (Guangzhou, China). Before transfection, cells were transplanted to 6-well or 96-well plates and grown to a confluence of 30%. Gp-transfect Mate (GenePharma, China) was used according to the manufacturer’s protocol. The ACSL4 plasmid was subcloned into the pcDNA3.1 vector to overexpress ACSL4. All plasmids were incubated with the transfection media for 48 h before OGD/R.

### 2.4 Lactate Dehydrogenase Assay and Cell Viability Assay

Serum and cellular supernatants were collected for the LDH activity assay (A020, JianCheng, China) according to the manufacturer’s instructions. The optical density value for each well at 450 nm absorbance was measured by Spectra Max 190 plate reader. Experiments were repeated at least three times.

To measure cell viability, 6 × 10^3^ cells per well were seeded in 96-well plates and treated as indicated, after which the medium in each well was replaced with 100 µL fresh medium containing 10% Cell Counting Kit-8 reagent (Apex BIO, K1018). After incubation for 1 h at 37°C according to the instructions of the kit, plate was read by a Spectra Max 190 plate reader at a wavelength of 450 nm.

### 2.5 Iron Measurements and Acyl-CoA Synthetase Long-Chain Family Member 4 Activity

Phosphate-buffered saline (PBS) was used for ischemic renal tissue homogenized. Collecting the supernatant after centrifugation. The content of iron was determined by the Iron Assay Kit (A039, JianCheng, China) according to the manufacturer’s instructions. ACSL4 was confirmed facilitate the esterification of arachidonoyl and adrenoyl into phosphatidylethanolamine (PE), a process closely related to ferroptosis (Kagan et al., 2017), so PE was used to evaluate the level of ACSL4 activity (Li et al., 2019). PE kits (ab241005, Abcam) were used according to the manufacturer’s instructions.

### 2.6 Lipid Peroxidation Assay

A lipid peroxidation assay kit (A106, Jiancheng, China) was used to test the lipid peroxidase (LPO) level in lysates of tissue following the manufacturer’s instructions. Briefly, lipid peroxide reacts with chromogenic reagents under the condition of 45°C for 60 min and produces a stable chromophore with a maximum absorption peak at 586 nm.

Lipid ROS was measured using the live-cell analysis reagent BODIPY 581/591 C11 (27,086, Cayman). Treated cells were incubated with BODIPY (5 μM) for 1 h at 37 °C in 24-well cultures. After incubation, cells were harvested and washed with PBS, and then resuspended in 500 μL PBS. Images were acquired under an IX83 fluorescence microscope (ECLIPSE Ts2R-FL, Nikon). Cell fluorescence was acquired on a Flow Cytometer (Cytomics^TM^FC500, Beckman) and analyzed with FlowJo software (FlowJo^TM^v10, United States).

### 2.7 Western Blot Analysis

The whole protein of mice renal tissues or HEK293T cells was extracted using the RIPA lysate buffer (Beyotime, China). Protein concentration was measured by a bicinchoninic acid reagent kit (Beyotime, China). The proteins were separated in 8% or 10% gels using a sodium dodecyl sulphate-polyacrylamide gel electrophoresis and then transferred onto polyvinylidene fluoride membranes (Millipore, Bedford, United States). The membranes were blocked with 5% non-fat milk at room temperature for 2 h and then incubated at 4°C overnight with the corresponding primary antibodies: GPx4 antibody (P02794, Abways), ACSL4 antibody (O60488, Abways); COX2 (ab62331, Abcam); β-tubulin antibody (86,298, Cell Signalling Technology). Next, the membranes were washed and incubated at room temperature for 2 h with the secondary antibodies: Goat anti-rabbit IgG antibody (CW0103S, CoWin), Goat anti-mouse IgG antibody (CW0102S, CoWin). The bands were detected using an enhanced chemiluinescence kit (NCM, China) under a luminescent imaging workstation (Tanon5200, China). The protein intensity was analyzed with the ImageJ software and normalized to β-tubulin.

### 2.8 Real-Time Quantitative Reverse Transcription-PCR (RT-qPCR)

The renal tissue was subjected to RNA extraction, reverse transcription, and then real-time quantitative reverse transcription-PCR as described before (Peng et al., 2020). The following primer sets (Sangon Biotech Inc, China) were used in the process: mouse ACSL4 (forward, 5′-CAA TAG AGC AGA GTA CCC TGA G-3′; reverse, 5′-TAG AAC CAC TGG TGT ACA TGA C-3′), mouse IL-6 (forward, 5′-TGA TGC ACT TGC AGA AAA CAA TCT GA-3′; reverse, 5′-AGC TAT GGT ACT CCA GAA GAC CAG AGG-3′), mouse TNF-α (forward, 5′-TGA TCG GTC CCC AA A GGG ATG; reverse, 5′-TTG GTG GTT TGC TAC GAC GTG G-3′), mouse β-tubulin (forward, 5′-CAG CGA TGA GCA CGG CAT AGA C; reverse, 5′-CCA GGT TCC AAG TCC ACC AGA ATG-3′). human IL-6 (forward, 5′-ATG AAC TCC TTC TCC ACA AGC GC -3′; reverse, 5′-GAA GAG CCC TCA GGC TGG ACT G -3′), human TNF-α (forward, 5′-TGA TCG GTC CCC AA A GGG ATG; reverse, 5′-TTG GTG GTT TGC TAC GAC GTG G-3′), human β-tubulin (forward, 5′-CAG CGA TGA GCA CGG CAT AGA C; reverse, 5′-CCA GGT TCC AAG TCC ACC AGA ATG-3′).

### 2.9 Transmission Electron Microscope (TEM)

After 48 h of reperfusion, the anesthetized mice were killed, the kidneys were washed with precooled PBS (pH 7.4), cut into 2 mm × 2 mm x 2 mm block tissue and then fixed in 100 mM PBS (pH 7.0–7.5) containing 2.5% glutaraldehyde (Servicebio, China) at room temperature for 2 h, and then transferred to 4°C for storage overnight. Ultrathin sections (70 nm) were cut, stained with uranyl acetate and lead citrate, and viewed under a transmission electron microscope (TEM; HT7700; Japan). Five fields for each sample was randomly chose and counted the mitochondria with ferroptotic features. The number of ferroptotic mitochondria per field in each sample was quantified.

### 2.10 GSH Assays and Malondialdehyde

Malondialdehyde (MDA) production was determined using a lipid peroxidation assay kit (A003, Jiancheng, China) based on the method of thiobarbituric acid reactive substances (TBARS) to reflect the degree of lipid peroxidation *in vivo*. Mice renal was homogenized and the supernatant was collected for GSH analysis using a GSH assay kit (A06, Jiancheng, China).

### 2.11 Assessment of Kidney Functions and Injury

Concentrations of serum creatinine and BUN were detected by Automatic biochemical instrument (Siemens ADVIA 1800 Chemistry System, Germany). Hematoxylin and Eosin (H&E)-stained kidney specimens were used to evaluate kidney injury, the renal sections were fixed in 4% paraformaldehyde and embedded in paraffin. Five millimeter slices were cut for H&E staining and then observed under an optical microscope (Olympus, Japan). Renal injury score was evaluated by an established grading by Zhou, W et al. as described before (Zhou et al., 2000). Renal injury was graded as fellow: normal (0 score); <10% necrosis (1score); 10–25% (2 score); 25–75% (3 score); and >75% (4).

### 2.12 Drug Treatment

Liproxstatin-1(Lip-1, B4987, APExBIO, United States), a ferrptosis inhibitor, was administrated i.p. at a concentration of 10 mg/kg 1 h before ischemia induction, in accordance with previous study protocols (Li et al., 2019). Rosiglitazone (ROSI, S2556,Selleck), a classic peroxisome proliferator-activated receptor-γ agonist that has been used for ACSL4 inhibition, was administered intravenously at a concentration of 0.4 mg/kg 1 h before ischemia induction (Doll et al., 2017; Li et al., 2019). Atipamezole (ATI, abs816081, absin), a α2-AR antagonist, was administered intraperitoneally at a concentration of 250 μg/kg 0.5 h before I/R treatment, and just the renal pedicle clamp was released. Dexmedetomidine (21,090,731, Yangtze River Pharmaceutical group, China) was administered intraperitoneally at a concentration of 50 μg/kg in two time points, including 30 min before I/R and just the renal pedicle clamp was released. Sham mice underwent all the procedures except renal ischemia. Blood and kidney samples were collected 48 h after reperfusion.

### 2.13 Statistical Analysis

Data were analyzed by GraphPad Prism 8.0 software (GraphPad Inc, La Jolla, United States) and expressed as means ± S.D. Statistical significance was determined by using Student’s *t*-test in two groups. ANOVA was used to compare more than two group. *p* < 0.05 was considered statistically significant.

## 3 Results

### 3.1 Dex Administration Improved Renal Function and Reversed Acyl-CoA Synthetase Long-Chain Family Member 4’s Upregulation During Renal I/R

**FIGURE 1:**
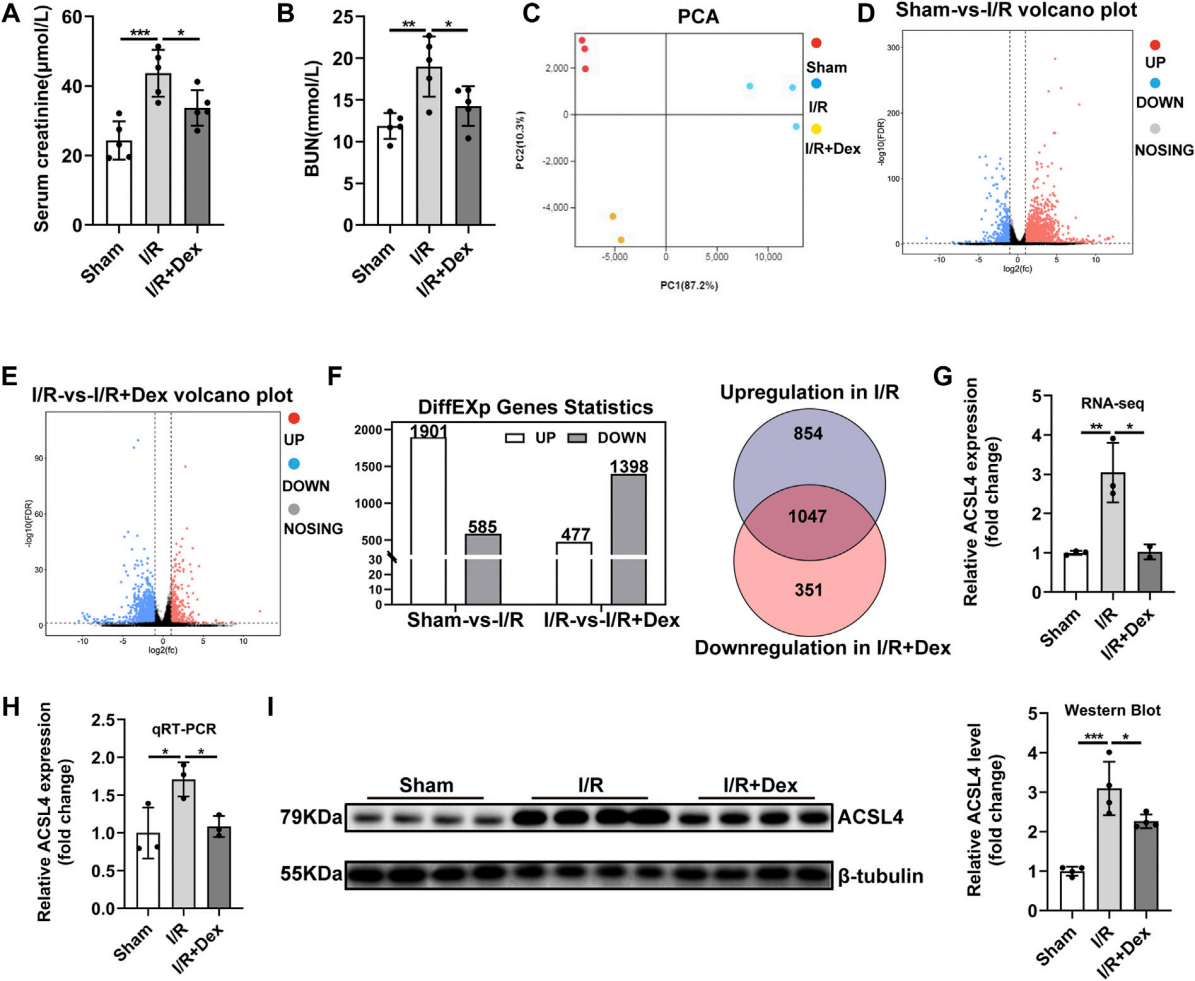
Dex administration improved renal function and reversed ACSL4 upregulation during renal I/R. **(A,B)** The Scr and BUN levels for assessing renal function (*n* = 5). **(C)** Principal component analysis (PCA) was performed. **(D–F)** Different expression genes statistics (ǀlog_2_FCǀ>1, and FDR<0.05). **(G)** Expression of ACSL4 gene in renal I/R tissue determined by RNA-sequencing (RNA-seq) (*n* = 3). **(H)** ACSL4 mRNA expression (n = 3). **(I)** ACSL4 protein expression (*n* = 4). The data are the Means ± S.D., *p < 0.05, **p < 0.01, ***p < 0.001.

The renal I/R injury significantly increased serum creatinine (Scr) and blood urea nitrogen (BUN) levels compared with the sham group, Dex administration effectively reduced the increased Scr and BUN levels (Figures 1A,B), which is consistent with the previous study that Dex has renal protection, but the exact protective mechanism is not clear, so we implemented RNA-seq to find the target gene of Dex. Principal component analysis (PCA) was performed with R package models (http://www.rproject.org/) in this experience. PCA is largely used to reveal the structure/relationship of the samples/datas (Figure 1C). The RNA-seq analysis showed 1901 up-regulated after renal I/R compared with the sham group, and 1398 down-regulated genes after Dex administration compared with the I/R group, Among them, 1047 genes have attracted our attention, as shown in the Venn diagram (ǀlog2FCǀ>1, and FDR<0.05, Figures 1D–F). We focused on the ACSL4, a key isozyme for polyunsaturated fatty acids metabolism that dictates ferroptosis sensitivity. RNA-seq result showed higher ACSL4 expression in the I/R group than those in the sham group and then significantly decreased with Dex administration **(** Figure 1G; Table1). The qRT-PCR (Figure 1H) and western blot (Figure 1I) results reveal that Dex significantly reversed the increased expression of ACSL4 following renal I/R injury.

**TABLE 1:** Top 15 up-regulated(Sham vs I/R)and down-regulated(I/R vs I/R + Dex)DEGs and ACSL4.

Symbol	Sham mean	I/R mean	log_2_(fc)	*p*-Value	FDR
Up-regulated genes(I/R vs Sham)					
Il1f6	0.001	4.753333	12.21472	3.78E-13	4.87E-12
Sprr2f	0.001	3.45	11.75238	3.94E-10	3.69E-09
Ccl20	0.001	3.163333	11.62723	3.85E-11	4.03E-10
Vgf	0.001	3.03	11.5651	3.45E-09	2.88E-08
1700001F09Rik	0.001	3.023333	11.56192	6.44E-11	6.59E-10
Sprr2g	0.001	2.36	11.20457	1.01E-08	7.96E-08
Gm45837	0.001	1.443333	10.49519	1.14E-14	1.69E-13
Crisp1	0.001	1.443333	10.49519	0.000263	0.001007
Il24	0.001	0.85	9.731319	3.42E-07	2.18E-06
Grp	0.001	0.846667	9.72565	2.26E-06	1.25E-05
Gm10375	0.001	0.816667	9.673604	3.75E-06	2.01E-05
Gm3486	0.001	0.68	9.409391	3.70E-05	0.000167
Cxcl17	0.001	0.626667	9.291554	9.61E-05	0.000401
Il6	0.001	0.573333	9.16323	1.63E-05	7.83E-05
Gsta1	0.066667	35.13667	9.041796	1.91E-14	2.77E-13
**Acsl4**	**10.77**	**34.5633**	**1.68222**	**3.37E-17**	**6.26E-16**
**Down-regulated genes(I/R + Dex vs I/R)**					
**Symbol**	**I/R mean**	**I/R + Dex mean**	**log**_**2**_ **(fc)**	***p*-Value**	**FDR**
Crisp1	1.443333	0.001	-10.4952	0.000844629	0.003877536
Gm20683	1.033333	0.001	-10.0131	1.47E-08	0.000000227
Samd1	0.92	0.001	-9.84549	0.000000881	0.00000924
Il24	0.85	0.001	-9.73132	0.0000131	0.000101867
Gm10375	0.816667	0.001	-9.6736	0.0000619	0.00039771
Kank2	0.696667	0.001	-9.44432	7.25E-08	0.000000964
Gm3486	0.68	0.001	-9.40939	0.000384486	0.001957323
Vgf	3.03	0.005	-9.24317	0.00000217	0.0000205
Arhgap36	0.56	0.001	-9.12928	0.00000124	0.0000125
Btbd17	0.386667	0.001	-8.59495	0.0000579	0.000375583
Gml	0.386667	0.001	-8.59495	0.00351278	0.013033653
Tmem59l	0.336667	0.001	-8.39518	0.001163536	0.005081411
Il5ra	0.29	0.001	-8.17991	0.0000738	0.000464371
Gcat	0.283333	0.001	-8.14636	0.00930505	0.029560402
Hmga1b	0.273333	0.001	-8.09452	0.010893458	0.03361304
**Acsl4**	34.56333	10.385	-1.73474	5.77E-11	1.46E-09

DEG, differentially expressed gene; FDR, false discovery rate.

### 3.2 Ferroptosis and Inflammation Are Involved in Renal Ischemia-Reperfusion

**FIGURE 2:**
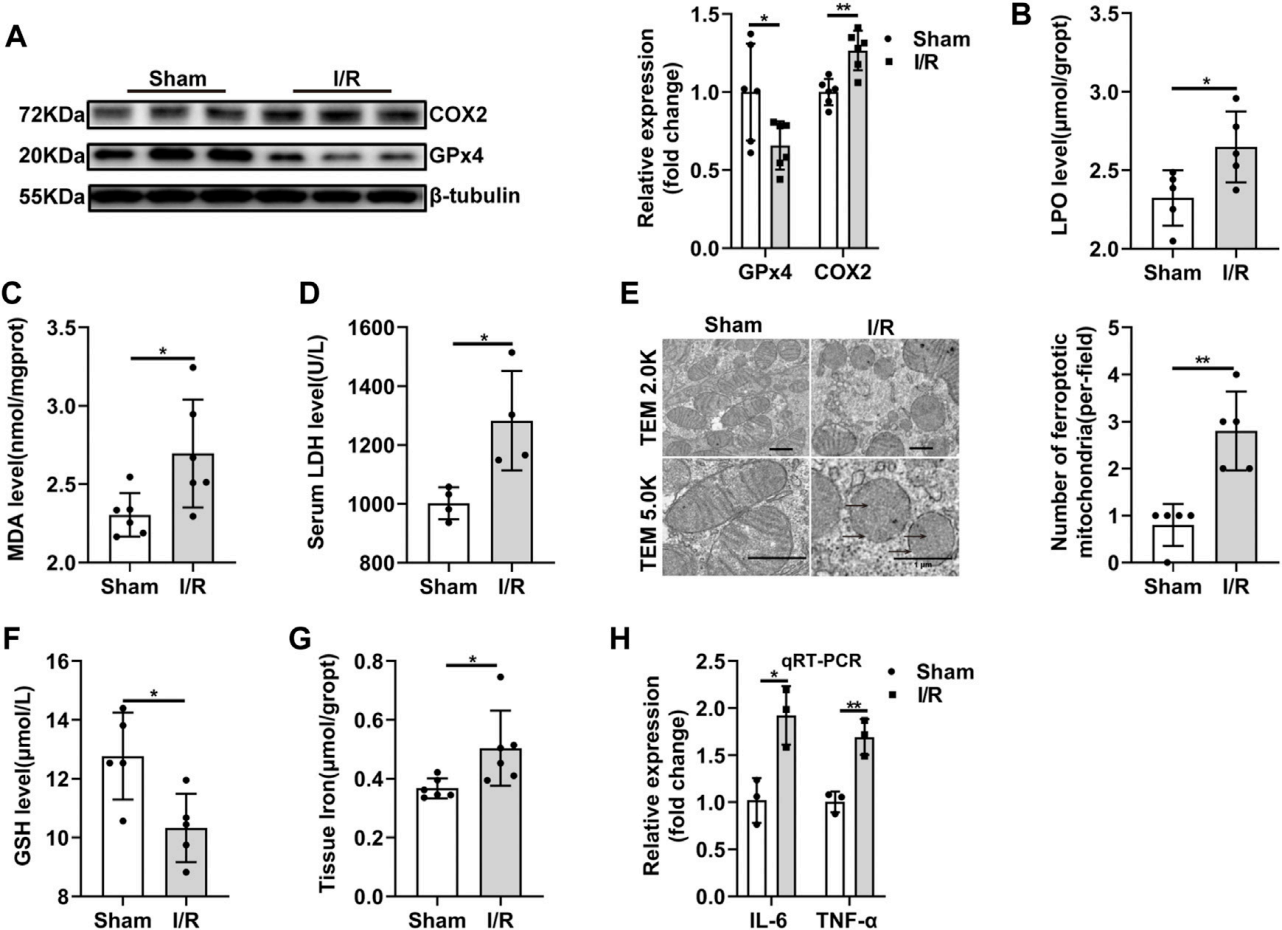
Ferroptosis and inflammation are involved in renal I/R. **(A)** Validation of GPx4 and COX2 protein expression by western blot analysis (*n* = 6). **(B–D)** LPO (*n* = 5), MDA (*n* = 6), LDH (*n* = 4) were measured from each group. **(E)** Representative TEM images (original magnification, × 2.0K or × 5.0K,scarle bar = 1 μm) and quantification were shown (*n* = 5). The black arrows indicate the decline or disappearance of mitochondrial cristae and the rupture of outer mitochondrial membrane **(F,G)** Tissue GSH (*n* = 5) and tissue iron content (n = 6) were measured from each group. **(H)** Validation of IL-6 mRNA, and TNF-α mRNA expression by qRT-PCR analysis (*n* = 3). The data are the Means ± S.D., *p < 0.05, **p < 0.01.

Some core factors, such GPx4, COX2, were confirmed as key and valid proteins in ferroptosis regulation (Li et al., 2019) (Chen et al., 2021). Therefore, to evaluate ferroptosis sensitivity after ischemia in the renal, we determined the expression levels of these proteins under I/R condition. The decreased GPx4 and increased COX2 protein expression in the I/R group were confirmed by western blot analysis (Figure 2A). LPO and MDA assay indicated that lipid peroxidation was higher in the I/R group than those in sham group (Figures 2B,C). LDH level was also increased after I/R injury (Figure 2D). TEM was used to investigate the morphological feature of ferroptosis. The mitochondrial morphological feature of ferroptosis was assessed, and the results showed that shrunk mitochondrial, reputed outer mitochondrial membrane, and the disappearance of mitochondrial cristae in the random field in the I/R group (Figure 2E). In addition, tissue GSH level was reduced in the I/R group (Figure 2F). And renal I/R led to an accumulation of tissue iron levels (Figure 2G). Our results also showed IL-6 mRNA and TNF-α mRNA, the critical indicators that involved in inflammation, were significantly increased during I/R (Figure 2H). Taken together, our result indicated that ferroptosis and inflammation may be contributed to renal I/R injury.

### 3.3 Inhibition of Ferroptosis Alleviates Renal Ischemia-Reperfusion Injury

**FIGURE 3:**
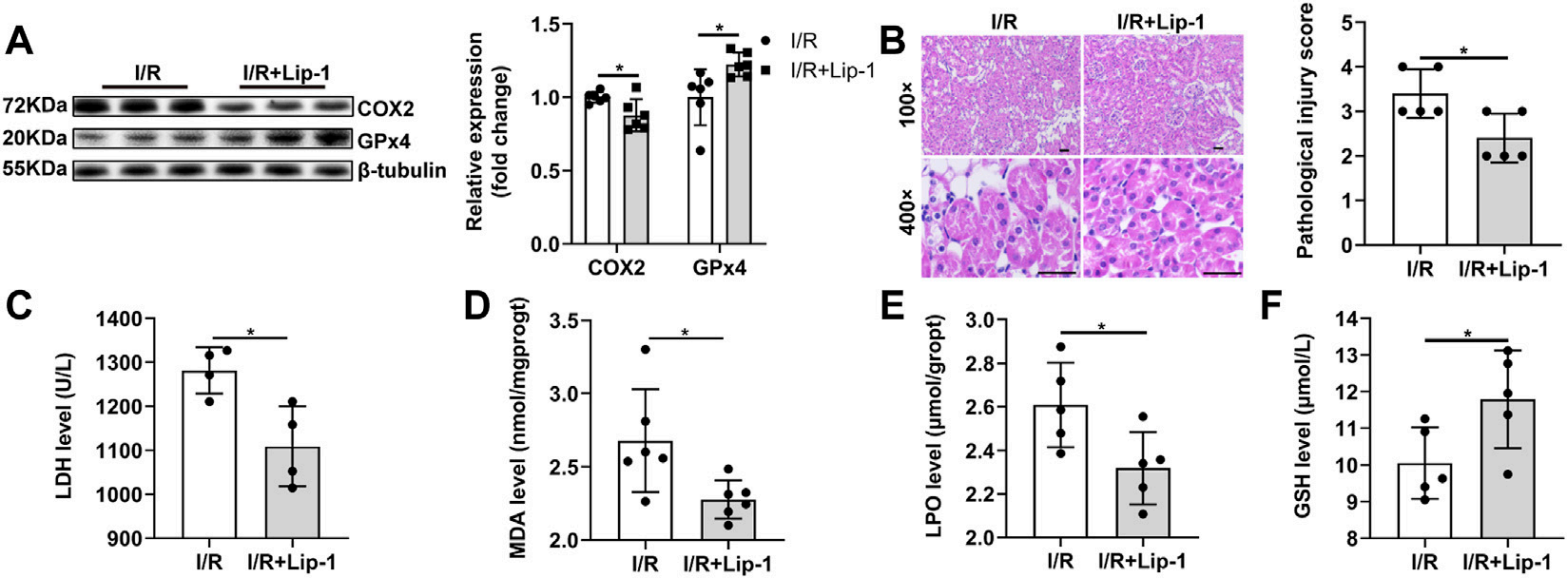
Liproxstatin-1(Lip-1) ameliorates renal I/R injury by inhibiting ferroptosis. **(A)** Western blot analysis of COX2 and GPx4 in the renal tissue (*n* = 6). **(B)** Representative H&E staining (original magnification, × 100 or × 400, scale bar = 20 μm) and corresponding renal injury score (n = 5). **(C)** Serum LDH level (*n* = 4). **(D)** MDA levels in renal homogenates (*n* = 6). **(E)** LPO production in renal homogenates (n = 5). **(F)** GSH levels in renal homogenates (*n* = 5). The data are the Means ± S. **(D)**, *p < 0.05.

In order to confirm the role of ferroptosis in renal I/R injury, Liproxstatin-1 (Lip-1), a specific ferroptosis inhibitor, was administrated. Lip-1 treatment significantly reversed the increased COX2 and decreased GPx4 protein expression induced by renal I/R injury (Figure 3A). The histopathological scores showed that renal I/R resulted in severe tubular damage, included widespread degeneration of tubular architecture, tubular dilation, tubular cell swelling, pyknotic nuclei, and luminal congestion, which could be significantly improved with Lip-1 treatment (Figure 3B). Lip-1 reduced the increased serum LDH level during I/R injury. (Figure 3C). Furthermore, Lip-1 also reduced MDA (Figure 3D) and LPO (Figure 3E), and increased the GSH levels (Figure 3F), indicating an improvement in lipid peroxidation and oxidation resistance.

### 3.4 Inhibition of Acyl-CoA Synthetase Long-Chain Family Member 4 Mitigates Ferroptosis-Mediated Damage and Inflammation

**FIGURE 4:**
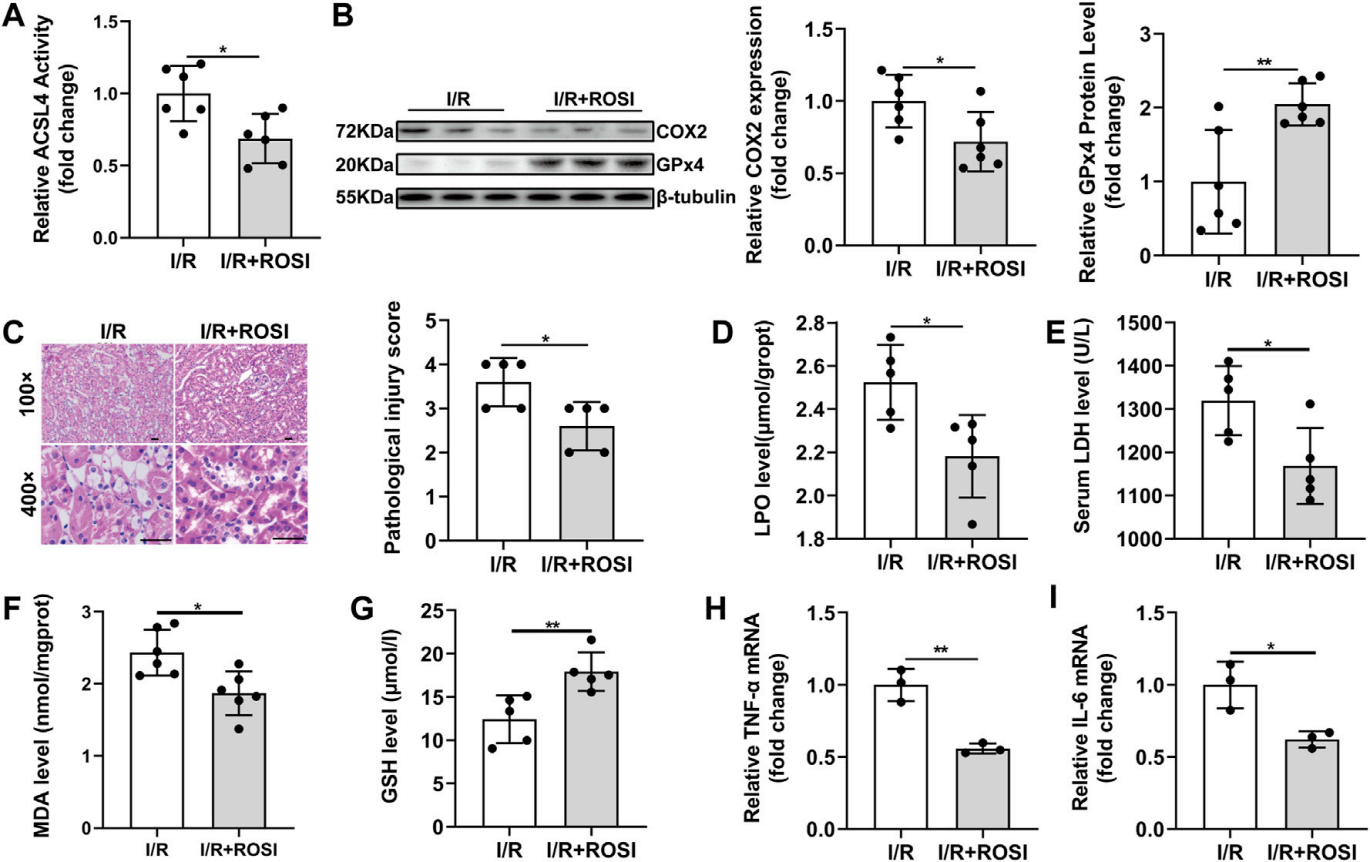
ROSI attenuates ferroptosis-mediated damage and inflammation during renal I/R. **(A)** ACSL4 activity (*n* = 6). **(B)** Western blot analysis of COX2 and GPx4 in the renal tissue (*n* = 6). **(C)** Representative H&E staining (original magnification, ×100 or × 400, scale bar = 20 μm) and corresponding relative renal injury score (*n* = 5). **(D)** LPO production (*n* = 5). **(E)** Serum LDH levels (*n* = 5). **(F–G)** MDA and GSH levels in renal homogenates (*n* = 5). **(H,I)** Expression of TNF-α mRNA and IL-6 mRNA from renal tissue of I/R determined by qRT-PCR analysis (*n* = 5). The data are the Means ± S.D., *p < 0.05, **p < 0.01.

ACSL4, a critical contributor and regulator of ferroptosis, determined the sensitivity of ferroptosis. Rosiglitazone (ROSI), a peroxisome proliferator-activated receptor-γ (PPAR-γ) activator, could inhibit ACSL4 activity. After ROSI treatment, ACSL4 activity was inhibited in the I/R + ROSI group compared with in the I/R group (Figure 4A). Meanwhile, the decreased GPx4 and increased COX2 protein expression in the I/R group were also reversed by ROSI treatment (Figure 4B). The relative histopathological score was significantly lower in the I/R + ROSI group than that in the I/R group (Figure 4C). ROSI treatment significantly decreased LPO, LDH and MDA level, as well as increased GSH level, in the kidney tissue during renal I/R injury (Figure 4D–G). Besides, the enhanced TNF-α mRNA and IL-6 mRNA levels induced by I/R were effectively reduced by ROSI treatment (Figures 4H,I). These results manifested that the suppression of ACSL4 activity alleviated ferroptosis-induced kidney damage and inflammation during renal I/R.

### 3.5 Acyl-CoA Synthetase Long-Chain Family Member 4 Overexpression Abolishes Dex-Mediated Protective Effects on OGD/R Induced HEK293T Cells Ferroptosis and Inflammation

**FIGURE 5:**
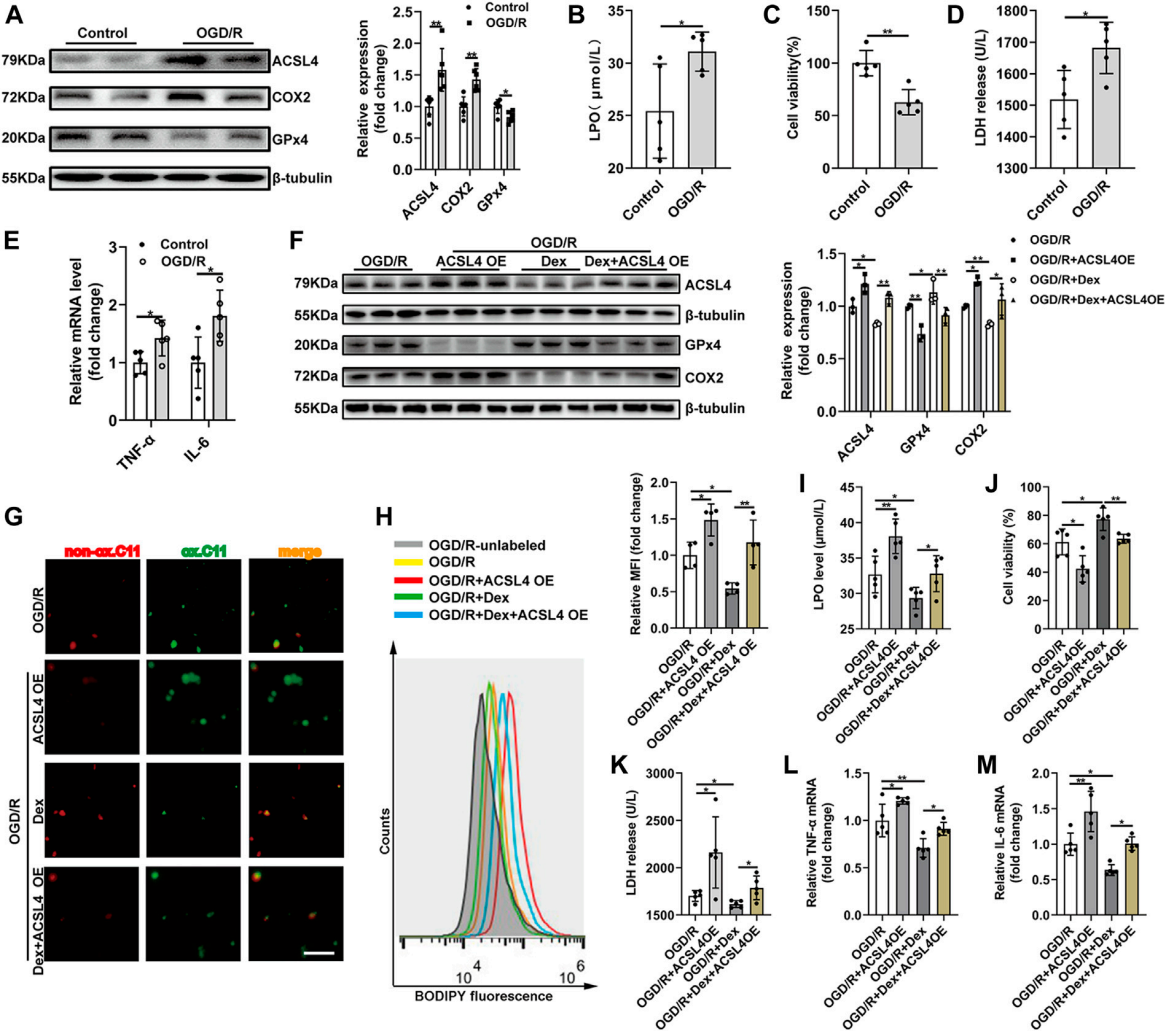
ACSL4 Overexpression Abolishes Dex-mediated Protective Effects on OGD/R Induced HEK293T Cells Ferroptosis and Inflammation. **(A)** Western blot was used to detect the ACSL4, COX2 and GPx4 protein expression in the HEK293T cell. The protein intensity was analyzed by using ImageJ (*n* = 6). **(B)** Cell lipid peroxidation were detected by LPO assay Kits (*n* = 5). **(C)** Cell survival was determined by CCK-8 kit after OGD/R (*n* = 5). **(D)** The level of LDH after OGD/R (*n* = 5). **(E)** The qRT-PCR analysis of IL-6 mRNA (*n* = 5) and TNF-α mRNA (*n* = 5). **(F)** The expression of ACSL4, Gpx4, and COX2 were detected by western blot (*n* = 3). **(G)** Cell survival was determined by CCK-8 kit (*n* = 5). **(H)**Cell lipid peroxidation were detected by BODIPY 581/591 C11 staining use fluorescence microscopy (original magnification, × 100, scale bar = 50 μm). **(I)** Representative histograms and Mean fluorescence intensity (MFI) of BODIPY oxidation in HEK293T cells (*n* = 4). **(J)** The level of released LDH (*n* = 5). **(K)** The level of LPO (*n* = 5). **(L)** The qRT-PCR analysis of TNF-α mRNA (*n* = 5). **(M)** IL-6 mRNA levels (*n* = 5). The data are the Means ± S.D., *p < 0.05, **p < 0.01.

To assess whether ACSL4 is the mediator of Dex exerting its effects, HEK293T cells were subjected to OGD/R. OGD/R significantly enhanced ACSL4 and COX2 expression and suppressed GPx4 expression (Figure 5A). OGD/R fortified the level of LPO (Figure 5B), suggesting that OGD/R contributes to lipid peroxidation. OGD/R increased cell death observed in the CCK-8 assay (Figure 5C). LDH release was also increased with OGD/R treatment (Figure 5D). In addition, OGD/R treatment increased inflammatory cytokines in HEK293T cells (Figure 5E). A distinct decrease in LDH release and increase in cell viability was observed in cells after lip-1 treatment (Supplementary Figure 1A, 1B). Then, HEK293T cells were transfected with ACSL4 overexpression plasmid for 48 h. ACSL4 overexpression effectively blocked the decreased ACSL4 in HEK293T cells during OGD/R with Dex treatment. Moreover, ACSL4 overexpression increased COX2 expression and decreased GPx4 expression (Figure 5F). As ACSL4 has been confirmed to aggrandize cell sensitivity to ferroptosis, we further evaluated the effects of ACSL4 on HEK293T cell ferroptosis. ACSL4 overexpression could partially reversed the protection of Dex on the inhibition of cell death determined by CCK-8 assay (Figure 5G). Dex decreased the density of green fluorescence represented by BODIPY581/591 C11 staining after OGD/R and moderately reversed by ACSL4 overexpression (Figure 5H). Lipid peroxidation analysis using BODIPY demonstrating that Dex significantly decreased cell ferroptosis and ACSL4 overexpression could reversed the protection of Dex to OGD/R-induced ferroptosis (Figure 5I). ACSL4 overexpression blocked the declined LDH (Figure 5J), and Dex effectively decreased lipid peroxidation as indicated by the decrease in LPO level (Figure 5K). Furthermore, ACSL4 overexpression abolished the decreased inflammatory cytokines (Figure 5L, 5M) in HEK293T cells during OGD/R with Dex treatment. These results demonstrated that Dex inhibited ferroptosis and inflammation induced by OGD/R, and this effect was partially reversed by ACSL4 overexpression, suggesting that Dex might regulate ferroptosis and inflammation by targeting ACSL4 during OGD/R.

### 3.6 Dex Attenuates Ferroptosis-Mediated I/R Injury and Inflammation by Downregulating Acyl-CoA Synthetase Long-Chain Family Member 4 Signaling *via* α2-AR

**FIGURE 6:**
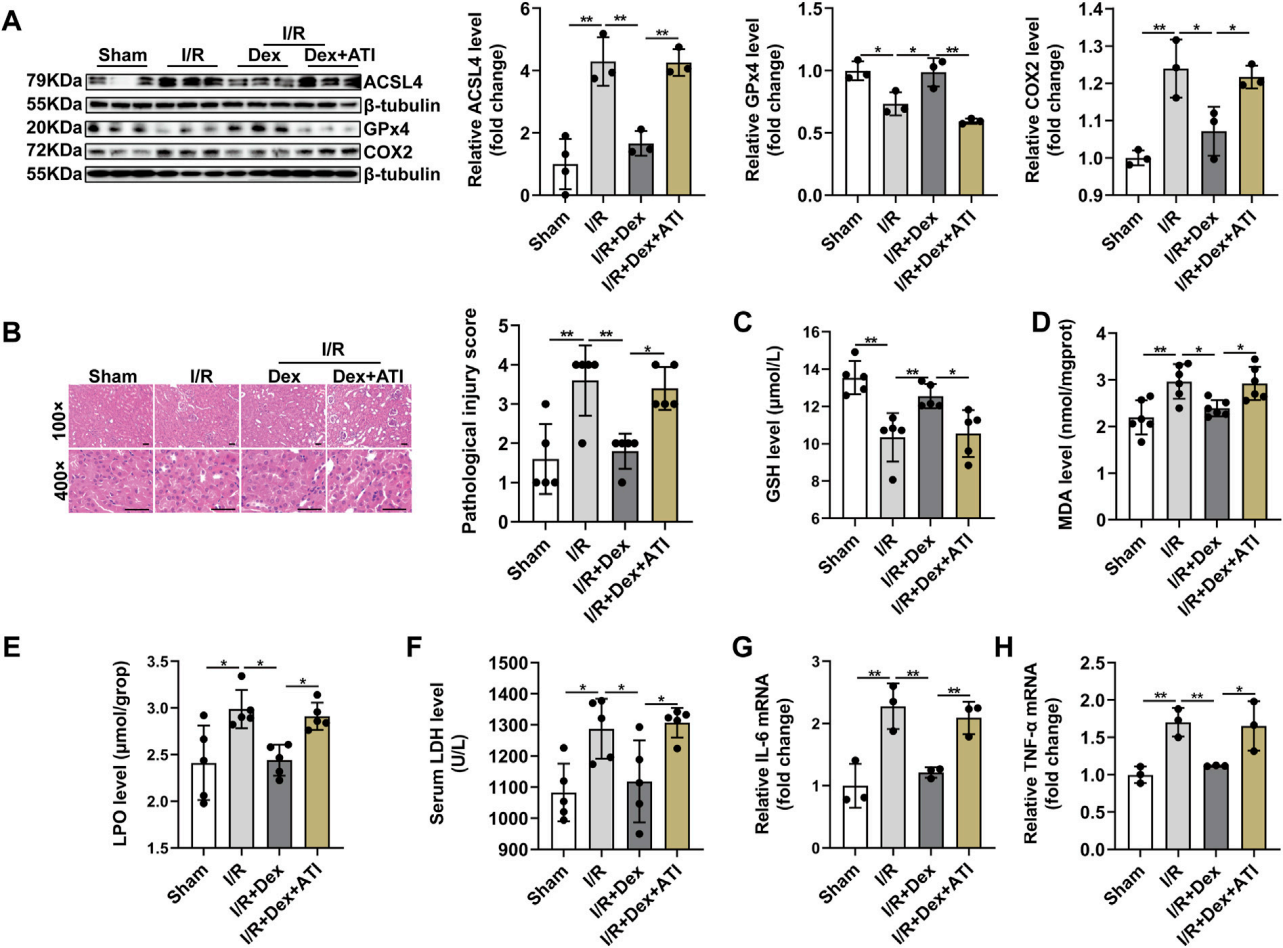
Dexmedetomidine alleviates ferroptosis-induced I/R injury and inflammation through targeting ACSL4 *via* α2-AR. **(A)** Validation of ACSL4, GPx4 and COX2 protein expression by western blot analysis (*n* = 6). **(B)** Representative H&E staining (original magnification, × 100 or × 400, Scale bar = 20 μm) and corresponding relative renal injury score were shown (*n* = 5). **(C–E)** GSH (*n* = 5), MDA (*n* = 6), LPO (*n* = 5) levels in renal homogenates (*n* = 5). **(F)** Serum LDH was measured from each group (*n* = 5). **(G,H)** Validation of IL-6 mRNA and TNF-α mRNA expression by qRT-PCR analysis (*n* = 5). The data are the Means ± S.D., *p < 0.05, **p < 0.01.

Base on the important role of ACSL4 signaling during I/R injury, we further investigated that whether ACSL4 signaling were involved in Dex-elicited protection against renal I/R injury. ATI administration effectively blocked the decreased ACSL4 and COX2 protein expression and increased GPx4 level suffering from Dex treatment during renal I/R injury (Figure 6A). ATI treatment declined the histopathological score (Figure 6B), as well as the reversion of increased GSH in kidney tissue induced by Dex treatment during I/R injury (Figure 6C). MDA production was higher in the I/R + Dex + ATI group than that in the I/R + Dex group (Figure 6D). ATI treatment significantly increased LPO and LDH levels (Figures 6E,F). ATI also attenuated the anti-inflammation effect of Dex (Figures 6G,H). In brief, ATI treatment significantly reversed the Dex effects on the inhibition role of ACSL4 during renal I/R injury, which suggest that Dex attenuates ferroptosis-mediated I/R injury and inflammation by downregulating ACSL4 signaling via α2-AR.

## 4 Discussions

Ischemia/reperfusion injury is a primary cause of AKI, which often arises from septic shock, major cardiovascular and abdominal surgeries, transplantation, and severe burns (Ergin et al., 2017; Jorge et al., 2019; Nieuwenhuijs-Moeke et al., 2020). Various clinical conditions lead to a decrease in renal blood flow, contributing to renal dysfunction and remote organ damage. Eventually, lead to multiple organ failure and death (Dépret et al., 2017). As the importance of I/R injury is becoming increasingly evident, the prevention of renal I/R injury has been a valid and reliable measure for AKI. Ferroptosis, as a type of regulated cell death, is different from other forms of cell death characterized by the accumulation of iron, lipid peroxidation, and condensed mitochondrial membrane densities. ACSL4 is a key enzyme that regulates lipid composition, has been shown to contribute to the execution of ferroptosis, but its role has not been widely investigated in renal I/R injury induced AKI. As a potent and highly selective α2-AR agonist, Dex has been widely used for long-term sedation and analgesia in an aesthesia because it induces a rapid response and is easily controllable. It is worth noting that some evidence observed in animal experiments indicated that Dex reduced kidney tissue damage and improved renal functional recovery during renal I/R injury (Si et al., 2013; Si et al., 2018). To the best of our current knowledge, this is the first study to demonstrate that Dex attenuates ferroptosis-mediated renal I/R injury and inflammation through inhibiting ACSL4 signaling via α2-AR.

Α2-adrenoceptors are widely distributed in distal and proximal tubules of the renal and peritubular vascular system (Kang et al., 2018; Bao and Dai, 2020). Studies have confirmed that Dex exerted a protective effects by reducing renal tubular damage and inhibiting apoptosis and inflammation in the tubular epithelial cells (Liang et al., 2017; Wang et al., 2020c). Dex pretreatment was also found to improve I/R renal microcirculation in experimental animals with I/R (Yang et al., 2021a). Renal I/R results in an increase in systemic and local sympathetic activity accompanied by intense vasoconstriction in the renal cortex. Therefore, studies have also confirmed that Dex alleviates renal lesion by resisting inflammatory of the sympathetic nervous system activation (Ma et al., 2020), improving outer renal medullary blood flow through local renal vasodilation(Billings et al., 2008), increasing glomerular filtration, dampening the ability of arginine vasopressin in the collecting duct, inhibiting the aquaporins along with the transport of Na^+^and water (Rouch et al., 1997), and alpha(2)-adrenoceptor agonist could regulate aquaporin-2 expression to stimulate urination (Junaid et al., 1999). Furthermore, Dex also brings down glomerular congestion, epithelial cell swelling, and stenosis in the luminal (Cakir et al., 2015). At the same time, the renal consists of several intrinsic cells, which injury or dysfunction can cause kidney damage. Therefore, more studies are needed to explore the effect of Dex on renal intrinsic cells and to comprehensively explain the renoprotective effect of Dex.

ACSL4, an important isozyme for polyunsaturated fatty acids (PUFAs) metabolism, has been identified as not only a sensitive regulator of ferroptosis but also an important contributor to the execution of ferroptosis (Doll et al., 2017; Xu et al., 2020). Inactivation of ACSL4 significantly alleviated the tissue damage in a mouse model of ferroptosis, suggesting that ACSL4 may be a target for ferroptosis inhibition (Angeli et al., 2017; Doll et al., 2017). Emerging evidence suggested that ferroptosis occurs in multiple organs under ischemia conditions, such as heart I/R injury (Wu et al., 2021a), renal I/R injury (Martin-Sanchez et al., 2020), intestinal I/R-induced lung injury (Ding et al., 2020) or even liver transplantation in the clinical setting (Yamada et al., 2020). One study found that ischemia-induced ACSL4 activation contributes to ferroptosis-mediated tissue injury in intestinal I/R (Li et al., 2019). And another study demonstrated that inhibiting ACSL4 mediated ferroptosis could prevent myocardial I/R injury (Fan et al., 2021). To date, the functional role of ACSL4 has rarely been reported in renal I/R-related diseases. Our present study found that ferroptosis was involved in the renal I/R injury, which was in line with the previous study (Su et al., 2019). Moreover, we found that ACSL4 expression was up-regulated after renal I/R injury and reversed by Dex administration. Besides, our results showed that the protective effect of Dex against renal I/R-induced injury was blocked by ACSL4 overexpression, revealing the important role of ACSL4 in the I/R injury. In this regard, ACSL4 may be a promising therapeutic target for alleviating ferroptosis-mediated renal I/R injury.

Ferroptosis, a non-apoptotic form of cell death, plays a detrimental role in I/R injury. Morphologically, ferroptosis mainly manifests as shrinkage of mitochondria, increased mitochondrial bilayer membrane density and reduction or disappearance of mitochondrial cristae, but the cell membrane is still intact. Biochemically, there is intracellular GSH depletion and inactivation of GPx4, lipid peroxides could not be metabolized by the GPx4-catalyzed reduction reaction, resulting in accumulation of ROS production, which triggers ferroptosis (Li et al., 2020b). And the shrunken mitochondria, increased lipid peroxidation and decreased GSH level was confirmed the occurrence of ferroptosis during renal I/R injury in our present study. Furthermore, Liproxstatin-1, the specific small-molecule inhibitor of ferroptosis, significantly attenuated ferroptosis-mediated renal I/R injury with decreased LPO level, reduced MDA level and increased GSH level. Besides, GPx4 serves a pivotal role in ferroptosis, and inhibition of GPx4 activity can lead to the accumulation of lipid peroxides. In GPx4 knockout mice, GPx4 deficiency leads to spontaneous acute renal failure and an increased rate of early mortality, whereas ferroptosis of renal tubular epithelial cells is the main cause of renal failure in GPx4 knockout mice (Friedmann Angeli et al., 2014). Yang and his colleagues demonstrated that COX2 is a suitable marker for the lipid peroxidation that occurs during GPx4-regulated ferroptosis (Yang et al., 2014). The results of the present study demonstrated that the GPX4 activity decreased, COX2 expression increased, and ferroptosis occurred during renal I/R injury in mice. Besides, inhibition of ACSL4 activity significantly reduced the COX2 and enhanced the GPx4 expression. Of note, upregulated COX2 expression has been found in the ferroptosis-induced mice (Yang et al., 2014). These findings indicated that the expression of COX2 might be regulated by ACSL4 activity during renal I/R injury.

Under normal physiological conditions, the resistance of cells and tissues to ferroptosis is higher than that of other forms of cell death (Stockwell et al., 2017). These results showed that the process of I/R changed the lipid metabolism, decreased the resistance of ferroptosis, and finally increased the incidence of ferroptosis in kidney tissue. It could not be ignored is that previous studies pointed out the important role of apoptosis, autophagy, and pyroptosis in renal I/R injury, as well as complex downstream mechanisms and signaling pathways (Fang et al., 2021; Xia et al., 2021; Xu et al., 2021). In addition, the critical role of inflammation in the development of renal I/R injury was confirmed in numerous studies (Qian et al., 2021; Reid and Scholey, 2021). In the precent study, we found that inhibition of ferroptosis or ACSL4 activity significantly decreased the IL-6 and TNF-α levels. And Dex administration attenuated renal I/R injury through suppressing ferroptosis and inflammation. However, we did not further explore the relationship between inflammation and ferroptosis. Clinical and experimental related study indicated that I/R refers to a complex inflammatory process that includes the synthesis of pro-inflammatory cytokines such as IL-6 and TNF-α, and the development of oxidative stress (Leurcharusmee et al., 2018). The oxidative stress caused by inflammatory responses further led to the initiation of lipid peroxidation, DNA damage, and mitochondrial function deterioration. And then further the development of ferroptosis (Gentile et al., 2017; Mao et al., 2020). In addition, Excessive iron is detrimental to the redox balance and can further enhance the production of inflammatory factors, leading to more damage (Gudjoncik et al., 2014).

In conclusion, the present study revealed that Dex exerted its protective effects partially by decreasing ferroptosis and inflammation through regulating ACSL4 via α2-AR. These results address the important role of Dex and ACSL4-mediated ferroptosis during renal I/R injury. DEX may be used as a therapeutic agent for patients with renal I/R-induced AKI.

## Data Availability

The datasets presented in this study can be found in online repositories. The names of the repository/repositories and accession number(s) can be found below: https://www.ncbi.nlm.nih.gov, PRJNA766819.
