# Supplementary material for: Dexmedetomidine Attenuates Ferroptosis-Mediated Renal Ischemia/Reperfusion Injury and Inflammation by Inhibiting ACSL4 via α2-AR
Source: Front Pharmacol. 2022 Jun 14;13:782466. doi: 10.3389/fphar.2022.782466 (PMC9307125; doi:10.3389/fphar.2022.782466)
Supplement: Supplementary file 1 [file Table1.DOCX]

**Table1. Top 15 up-regulated（Sham vs. I/R）and down-regulated（I/R vs. I/R+Dex）DEGs and ACSL4.**

| **Symbol** | **Sham mean** | **I/R mean** | **log_2_(fc)** | ***p*-Value** | **FDR** |
| --- | --- | --- | --- | --- | --- |
| **Up-regulated genes（I/R vs. Sham）** | | | |  |  |
| Il1f6 | 0.001 | 4.753333 | 12.21472 | 3.78E-13 | 4.87E-12 |
| Sprr2f | 0.001 | 3.45 | 11.75238 | 3.94E-10 | 3.69E-09 |
| Ccl20 | 0.001 | 3.163333 | 11.62723 | 3.85E-11 | 4.03E-10 |
| Vgf | 0.001 | 3.03 | 11.5651 | 3.45E-09 | 2.88E-08 |
| 1700001F09Rik | 0.001 | 3.023333 | 11.56192 | 6.44E-11 | 6.59E-10 |
| Sprr2g | 0.001 | 2.36 | 11.20457 | 1.01E-08 | 7.96E-08 |
| Gm45837 | 0.001 | 1.443333 | 10.49519 | 1.14E-14 | 1.69E-13 |
| Crisp1 | 0.001 | 1.443333 | 10.49519 | 0.000263 | 0.001007 |
| Il24 | 0.001 | 0.85 | 9.731319 | 3.42E-07 | 2.18E-06 |
| Grp | 0.001 | 0.846667 | 9.72565 | 2.26E-06 | 1.25E-05 |
| Gm10375 | 0.001 | 0.816667 | 9.673604 | 3.75E-06 | 2.01E-05 |
| Gm3486 | 0.001 | 0.68 | 9.409391 | 3.70E-05 | 0.000167 |
| Cxcl17 | 0.001 | 0.626667 | 9.291554 | 9.61E-05 | 0.000401 |
| Il6 | 0.001 | 0.573333 | 9.16323 | 1.63E-05 | 7.83E-05 |
| Gsta1 | 0.066667 | 35.13667 | 9.041796 | 1.91E-14 | 2.77E-13 |
| **Acsl4** | **10.77** | **34.5633** | **1.68222** | **3.37E-17** | **6.26E-16** |
|  |  |  |  |  |  |
| **Down-regulated genes（I/R+Dex vs. I/R）** | | | |  |  |
| **Symbol** | **I/R mean** | **I/R+Dex mean** | **log_2_(fc)** | ***p*-Value** | **FDR** |
| Crisp1 | 1.443333 | 0.001 | -10.4952 | 0.000844629 | 0.003877536 |
| Gm20683 | 1.033333 | 0.001 | -10.0131 | 1.47E-08 | 0.000000227 |
| Samd1 | 0.92 | 0.001 | -9.84549 | 0.000000881 | 0.00000924 |
| Il24 | 0.85 | 0.001 | -9.73132 | 0.0000131 | 0.000101867 |
| Gm10375 | 0.816667 | 0.001 | -9.6736 | 0.0000619 | 0.00039771 |
| Kank2 | 0.696667 | 0.001 | -9.44432 | 7.25E-08 | 0.000000964 |
| Gm3486 | 0.68 | 0.001 | -9.40939 | 0.000384486 | 0.001957323 |
| Vgf | 3.03 | 0.005 | -9.24317 | 0.00000217 | 0.0000205 |
| Arhgap36 | 0.56 | 0.001 | -9.12928 | 0.00000124 | 0.0000125 |
| Btbd17 | 0.386667 | 0.001 | -8.59495 | 0.0000579 | 0.000375583 |
| Gml | 0.386667 | 0.001 | -8.59495 | 0.00351278 | 0.013033653 |
| Tmem59l | 0.336667 | 0.001 | -8.39518 | 0.001163536 | 0.005081411 |
| Il5ra | 0.29 | 0.001 | -8.17991 | 0.0000738 | 0.000464371 |
| Gcat | 0.283333 | 0.001 | -8.14636 | 0.00930505 | 0.029560402 |
| Hmga1b | 0.273333 | 0.001 | -8.09452 | 0.010893458 | 0.03361304 |
| **Acsl4** | **34.56333** | **10.385** | **-1.73474** | **5.77E-11** | **1.46E-09** |

**DEG, differentially expressed gene; FDR, false discovery rate.**
