# Supplementary figures and images for: Dexmedetomidine Attenuates Ferroptosis-Mediated Renal Ischemia/Reperfusion Injury and Inflammation by Inhibiting ACSL4 via α2-AR
Source: Front Pharmacol. 2022 Jun 14;13:782466. doi: 10.3389/fphar.2022.782466 (PMC9307125; doi:10.3389/fphar.2022.782466)

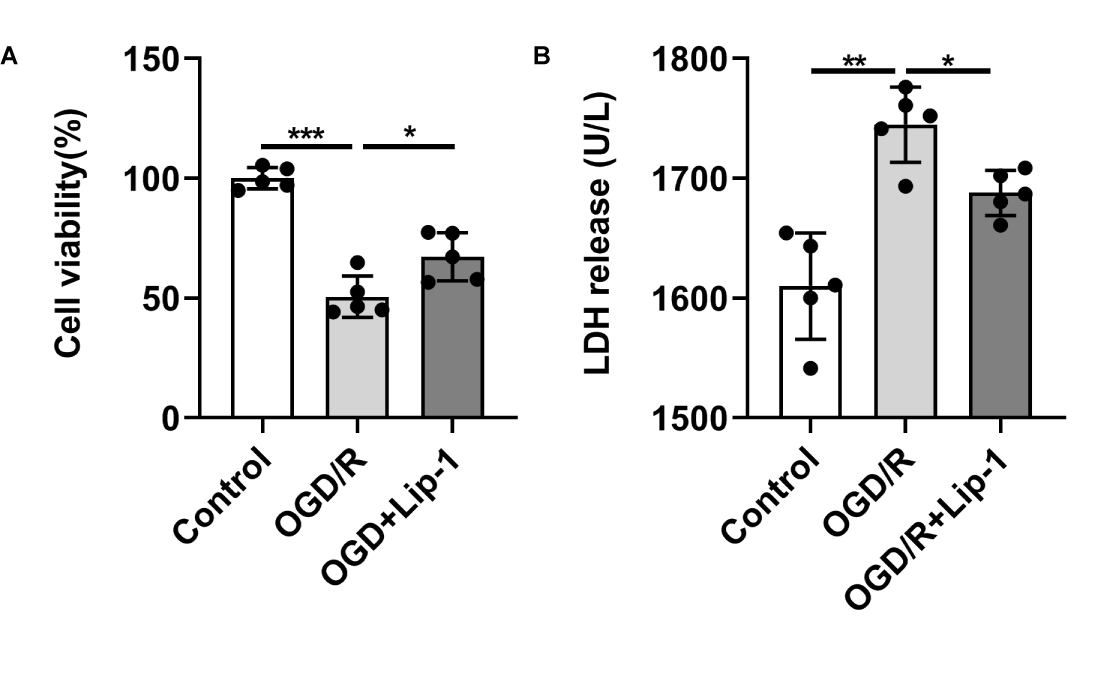

Supplement: Supplementary file 2 [file Image1.TIF]
